# Supplementary material for: Crystal structures of the elusive Rhizobium etlil-asparaginase reveal a peculiar active site
Source: Nat Commun. 2021 Nov 18;12:6717. doi: 10.1038/s41467-021-27105-x (PMC8602277; doi:10.1038/s41467-021-27105-x)
Supplement: Supplementary file 1 — Supplementary Information [file 41467_2021_27105_MOESM1_ESM.pdf]

Supplementary Information: *Crystal structures of the elusive Rhizobium etli L-asparaginase reveal a peculiar active site*

Supplementary Tables

Supplementary Table 1. Sequences of mutagenic primers (Q5 site directed mutagenesis). Mutation sites are underlined.

Mutant	Forward primer	Reverse primer
S48A	GCTCGCCCGGGCTGCGGCGAAGC	GTCATACGCGTCGGATTTCCCAGCGCGTAGAG
K51A	GTCTGCGGCGGCGCCGGCGCAG	CGGGCGAGCGTCATACGC
S80A	GATGTGCGCGGCCCACAGCAG	AGTGCAATATCCGCATCATCGAAGC
C135A	TTGCAGCAATGCCTCGGGCAAGC	ACCGCCGTCGGGATAAAG
K263A	CCTCGTCGGCGCGCTCGGGGCCG	GCGCCGTCGAATGCGCGC

Supplementary Table 2. Data collection and structure refinement statistics

Structure	START	OP	MP1	MP2	MC
**Data collection**					
Beamline/radiation source	P13/Petra III, DESY, Hamburg	P13/Petra III, DESY, Hamburg	P13/Petra III, DESY, Hamburg	BL14.3/BESSY II, Berlin	BL14.1/BESSY II, Berlin
Wavelength (Å)	2.0664	0.9762	0.9763	0.8950	0.9184
Temperature (K)	100	100	100	100	100
Space group	$P2_12_12_1$	$P2_12_12_1$	$P2_1$	$P2_1$	$C2$
Unit cell parameters<br>a, b, c (Å)<br>α, β, γ (º)	 78.15, 91.05, 105.84<br>90, 90, 90	 78.23, 91.14, 106.02<br>90, 90, 90	 77.81, 91.04, 113.66<br>90, 97.1, 90	 77.92, 91.31, 114.16<br>90, 97.1, 90	 130.29, 87.28, 93.93<br>90, 130.2, 90
Oscillation range (º)	0.10	0.25	0.20	1.00	0.10
No. of images	14400 /4x3600/	1440	1100	200	3600
Resolution range (Å)	105.84 - 2.18 (2.23 - 2.18)[a]	69.11 - 1.29 (1.40 - 1.29)	77.22 - 1.43 (1.52 - 1.43)	45.65 - 1.65 (1.75 - 1.65)	46.73 - 1.76 (1.86 - 1.76)
Reflections collected/unique	1340667 / 75182[b]	1663737 / 131038	1174400 / 287575	764746 / 189888	532099 / 78980
Completeness (%)	98.3 (79.4)	69.2 (spherical)<br>95.4 (ellipsoidal)	99.3 (96.8)	99.0 (98.0)	98.3 (95.7)
Multiplicity	17.8 (4.1)	12.7 (10.4)	4.1 (3.4)	4.0 (4.1)	6.7 (6.6)
R_{merge} (%)	8.8 (69.4)	17.1 (200.8)	7.3 (81.8)	13.4 (62.2)	4.2 (55.6)
Wilson B-factor ($Å^2$)	37.21	19.77	22.93	21.54	36.41
$\langle I/\sigma(I)\rangle$	21.7 (2.1)	10.4 (1.5)	10.2 (1.5)	10.0 (2.2)	22.0 (2.7)
$CC_{1/2}$	99.9 (84.6)	99.6 (40.0)	99.8 (67.1)	99.5 (79.8)	100.0 (92.5)
**Refinement**					
Protein chains in ASU	2	2	4	4	2
Matthews vol. ($Å^3$/Da) / solvent (%)	2.47 / 50.3	2.40 / 49.4	2.60 / 51.2	2.60 / 52.6	2.61 /53.0
Unique / test reflections	38687 / 1000	128695 / 1000	286572 / 1000	188888 / 1000	77974 / 1000
R_{work} / R_{free} (%)	16.9 / 22.8	15.6 / 19.7	15.8 / 17.9	18.9 / 22.2	18.3 / 22.7
Protein / solvent atoms	5323 / 314	5362 / 622	10352 / 1543	10372 / 1403	5400 / 333
$\langle B\rangle$ ($Å^2$) protein / solvent	36.23 / 53.33	14.31 / 33.01	19.20 / 34.26	16.80 / 27.42	35.19 / 50.99
Rmsd bonds (Å) / angles (º)	0.008 / 1.424	0.014 / 1.801	0.011 / 1.681	0.013 / 1.645	0.015 / 1.652
Ramachandran plot (%)<br>favored/allowed/outliers	96 / 4 / 0	98 / 2 / 0	97 / 3 / 0	98 / 2 / 0	97/ 3 /0
**PDB code**	**7os3**	**7os5**	**7os6**	**7ou1**	**7oz6**

[a]Values in parentheses correspond to the last resolution shell.
[b]Anomalous scaling (Bijvoet pairs separate) for S-SAD calculations.

Supplementary figures

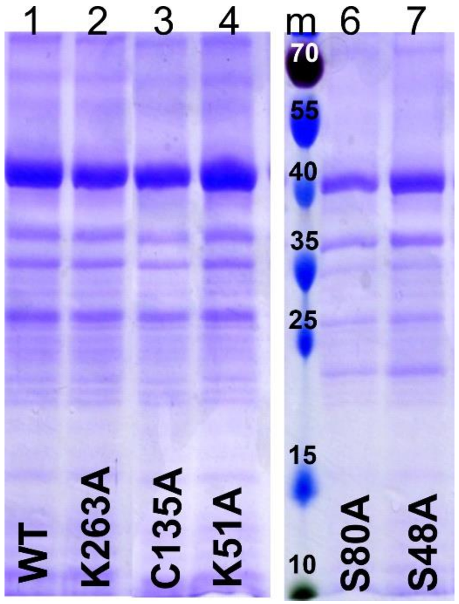

Supplementary Figure 1. SDS-PAGE gels showing expression levels (whole cell lysates) of WT and mutant ReAV. WT ReAV (lane 1, 38.9 kDa) and its mutants: S48A (lane 7), K51A (lane 4), S80A (lane 6), C135A (lane 3) and K263A (lane 2). Numbers in lane m show the molecular weight of mass markers in kDa. The expression pattern was reproducible in multiple protein purification runs.

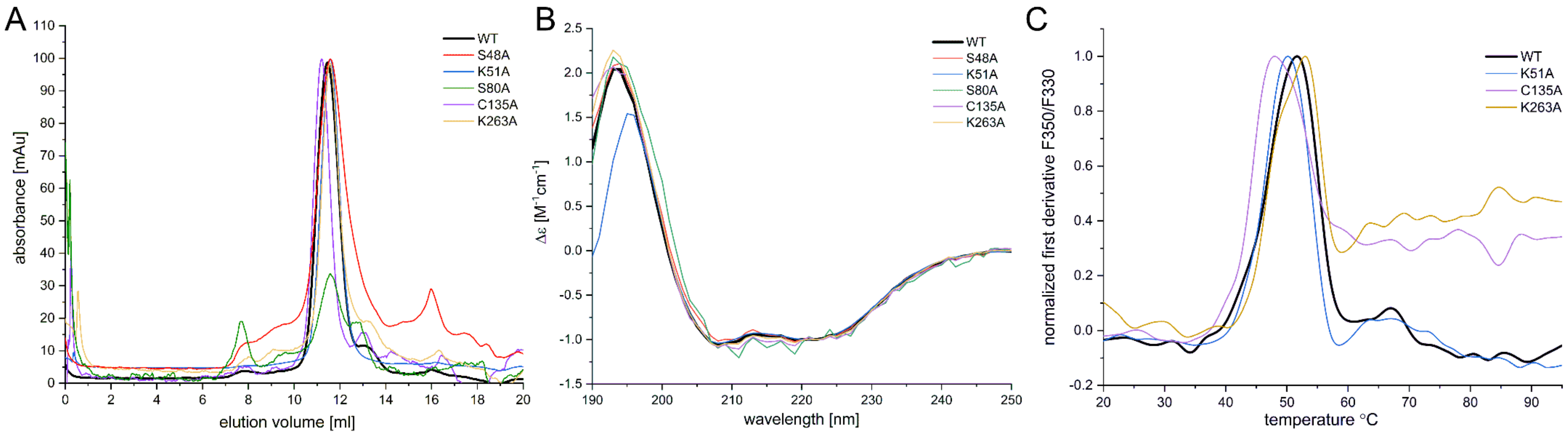

Supplementary Figure 2. FPLC chromatograms, CD spectra, and nanoDSF plots for WT ReAV and its mutants. (A) Elution profiles (*Superdex 10/300 GL*) of WT ReAV and its five mutants. Fractions from the top of the elution peak were collected and subjected to (B) CD studies; the plotted spectra for different variants were normalized to the same Δε value at 222 nm. (C) NanoDSF thermal stability measurements; the denaturation of S48A and S80A could not be reliably measured (data not shown). Source data are provided as a Source Data file.

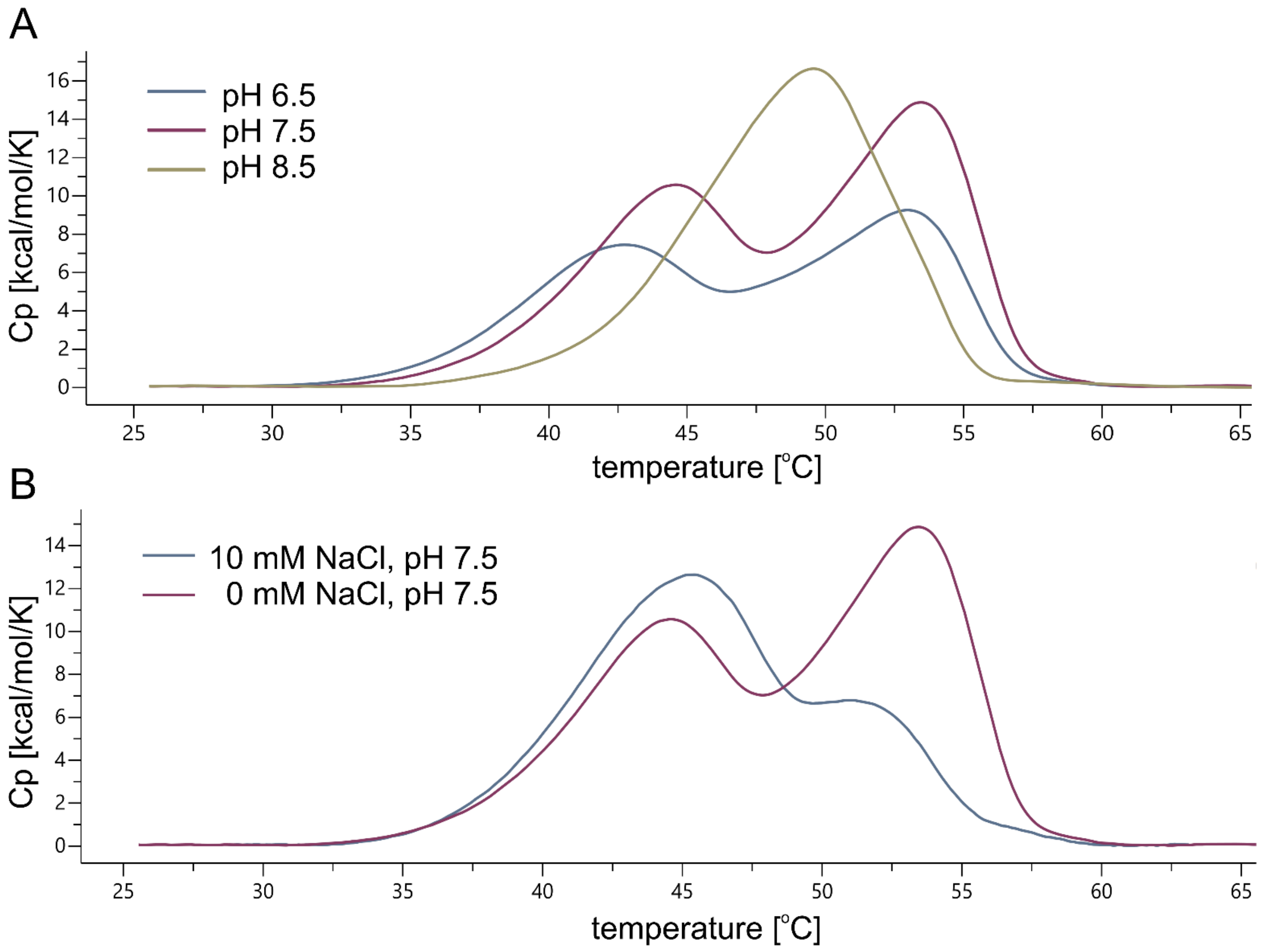

Supplementary Figure 3. Thermal unfolding of WT ReAV monitored by DSC. (A) Results obtained in buffers of different pH. (B) Melting curves recorded at pH 7.5 in the absence and presence of 10 mM NaCl. Cp is heat capacity measured in the units of [kcal/mol/K]. Source data are provided as a Source Data file.

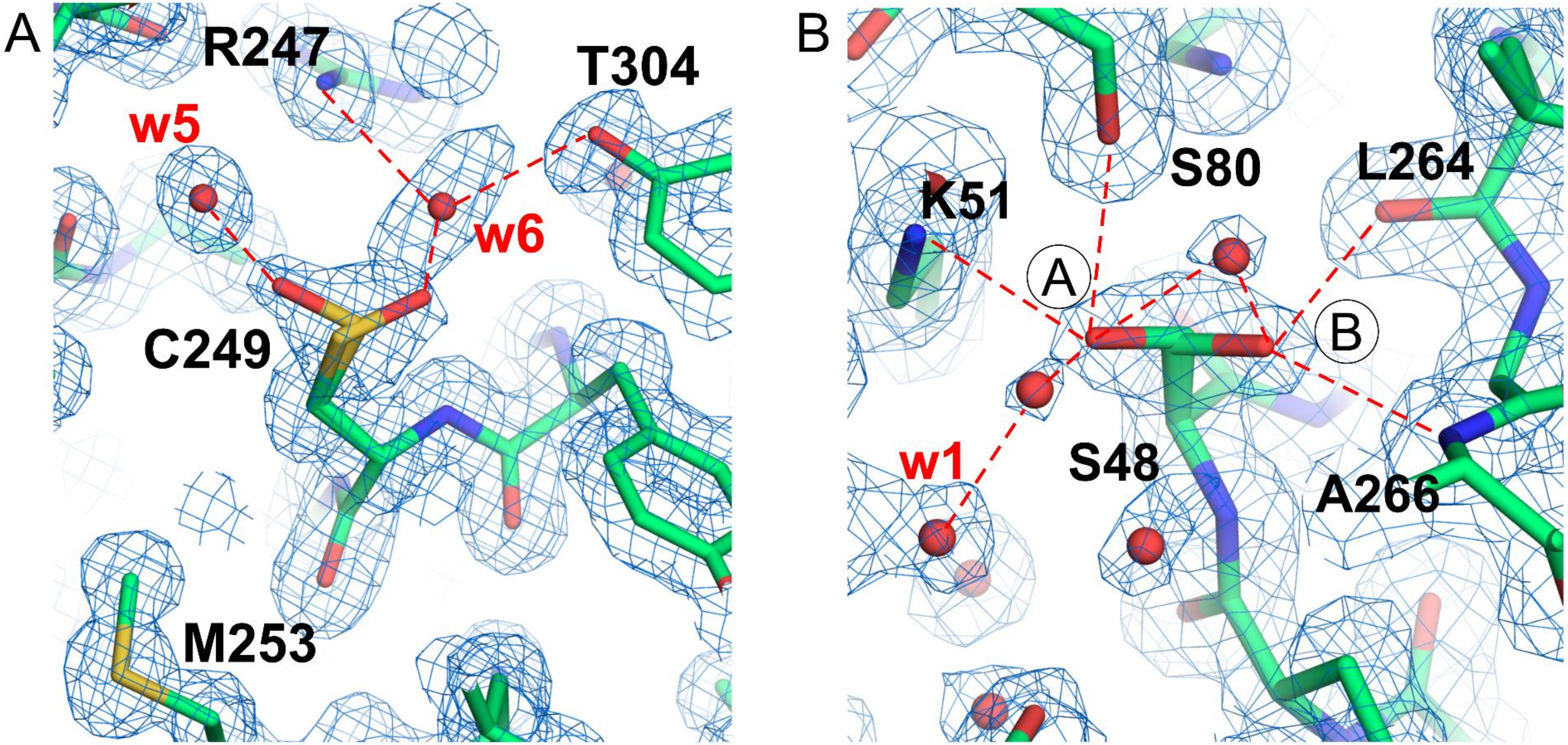

Supplementary Figure 4. 2Fo-Fc electron density maps (contour levels 1.20σ) around selected residues. (A) Cys249 carrying a modification (oxidation), as seen in structure **OP** (1.29 Å). (B) double-conformation Ser48 in structure **MP1** (1.65 Å). In panel (B) the alternative conformations of Ser48 are marked as "A" and "B". Water molecules are shown as red spheres, H-bonds are represented by dashed lines.

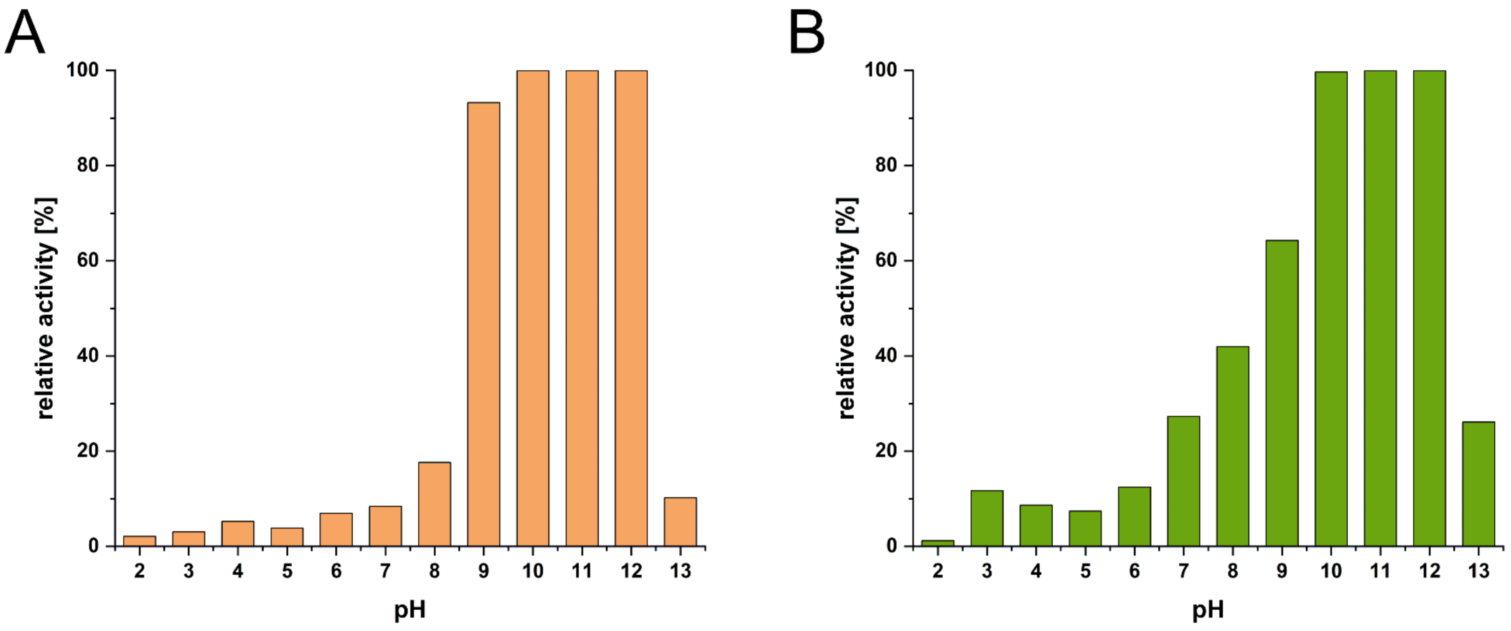

Supplementary Figure 5. pH profile of ReAV L-asparaginase activity in Britton-Robinson buffers. Maximum activity (100%), measured as maximum optical density/absorbance at a given wavelength, was observed at pH 10-12 using both (A) the Nessler method (measurement of optical density at 420 nm) and (B) Berthelot method (measurement of absorbance at 640 nm). In both assays, L-asparaginase activity was assessed using 2.2 µg of WT enzyme, 40 mM of L-Asn substrate, and 20-min incubation. Source data are provided as a Source Data file.

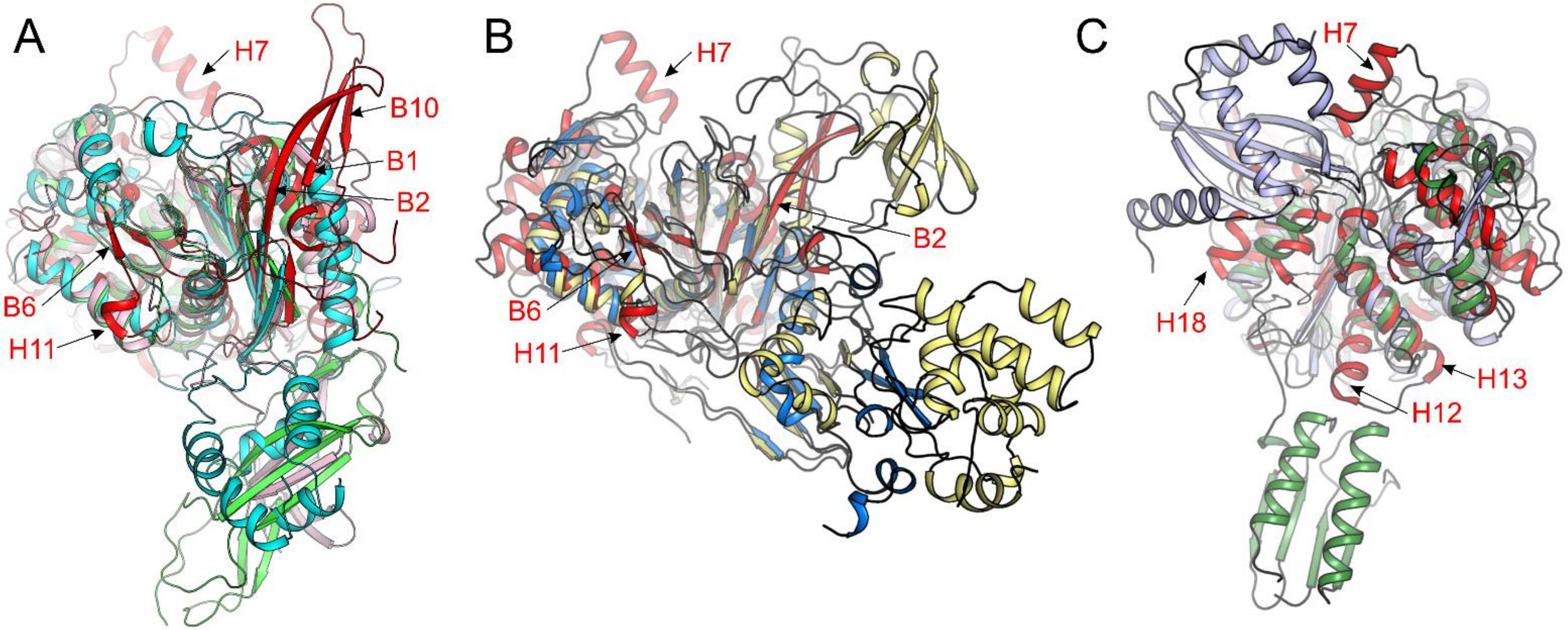

Supplementary Figure 6. Superposition of ReAV (red) and its structural homologs possessing additional domains. In all examples ReAV superposes quite well on the large catalytic domain of the matches. (A) ReAV superposition on PBP from *S. aureus* (light pink, 6c39), PBP from *E. coli* (green, 1zno) and kidney-type mouse glutaminase (cyan, 4jkt). (B) ReAV superposition on PBP from *A. baumannii* (light yellow, 3ue1), peptidoglycan glycosyltransferase from *A. parvulum* (blue, 4r1g). (C) ReAV superposition on glutaminase from *M. luteus* (dark green, 3if5) and β-lactamase from *S. clavuligerus* (light violet, 2xgn). Structural elements that differentiate ReAV from the other proteins are marked by arrows. Red sphere is a position of the zinc ion coordinated in the ReAV active sites.

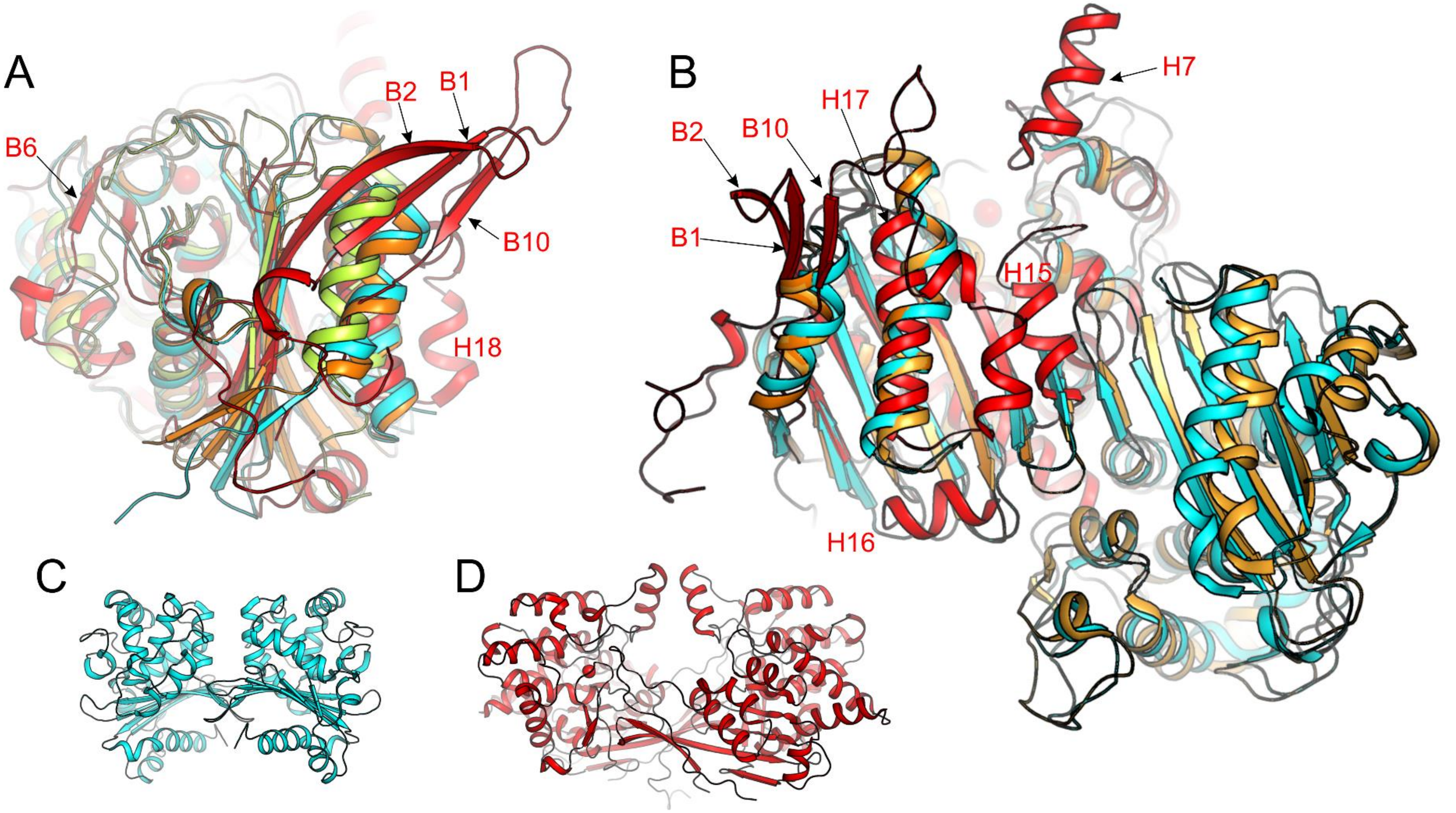

Supplementary Figure 7. Superposition of ReAV (red) with selected structural homologs. (A) Superposition of ReAV with selected β-lactamases from: *E. coli* (orange, 3qnc), *K. pneumoniae* (cyan, 6v1o) and *P. aeruginosa* (light green, 5eua). In the position corresponding to ReAV β-strands B1, B2 and B10, an α-helix is present in the other proteins. (B) Superposition of β-lactamase dimers form *E. coli* (orange) and *K. pneumoniae* (cyan) with ReAV dimer. The β-lactamase dimers are formed by interactions of antiparallel β-strands from two subunits, while ReAV dimerizes in an entirely different way, via strands B1, B2, B10, and helix H7. The structural elements that differentiate ReAV from the other proteins are marked by arrows. The red sphere marks the position of the zinc ion coordinated in the ReAV active site. Comparison of the dimeric structure of β-lactamase (6v1o) (C) and ReAV (D) is shown at the bottom left.

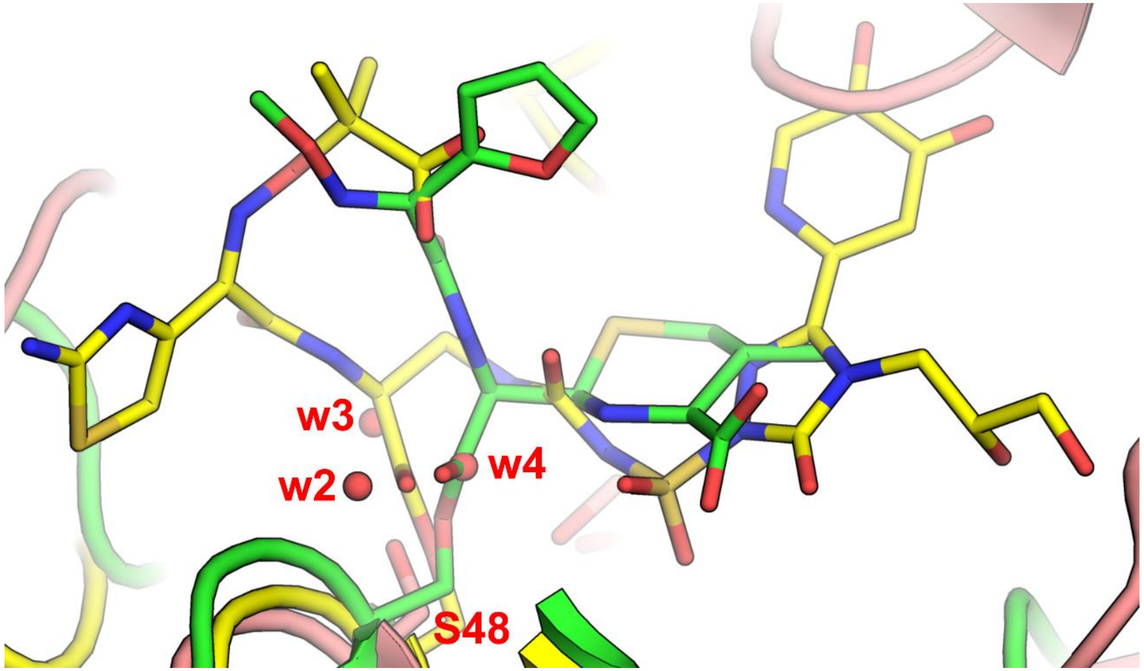

Supplementary Figure 8. Structural interpretation of the hydration water triad around Ser48. The water (red balls) triad w2-w3-w4 near Ser48 in ReAV (salmon) might resemble the tetrahedral transition state of the enzymatic reaction, as in the corresponding region of its structural homologs (identified by their PDB codes and color), covalently bound antibiotics were found. Shown in structural superposition are penicillin binding protein (PBP) from *A. baumannii* with covalently bound siderophore-conjugated monocarbam MC-1 (yellow, 3ue1), and PBP from *L. monocytogenes* with covalently attached cefuroxime (green, 5zqe).

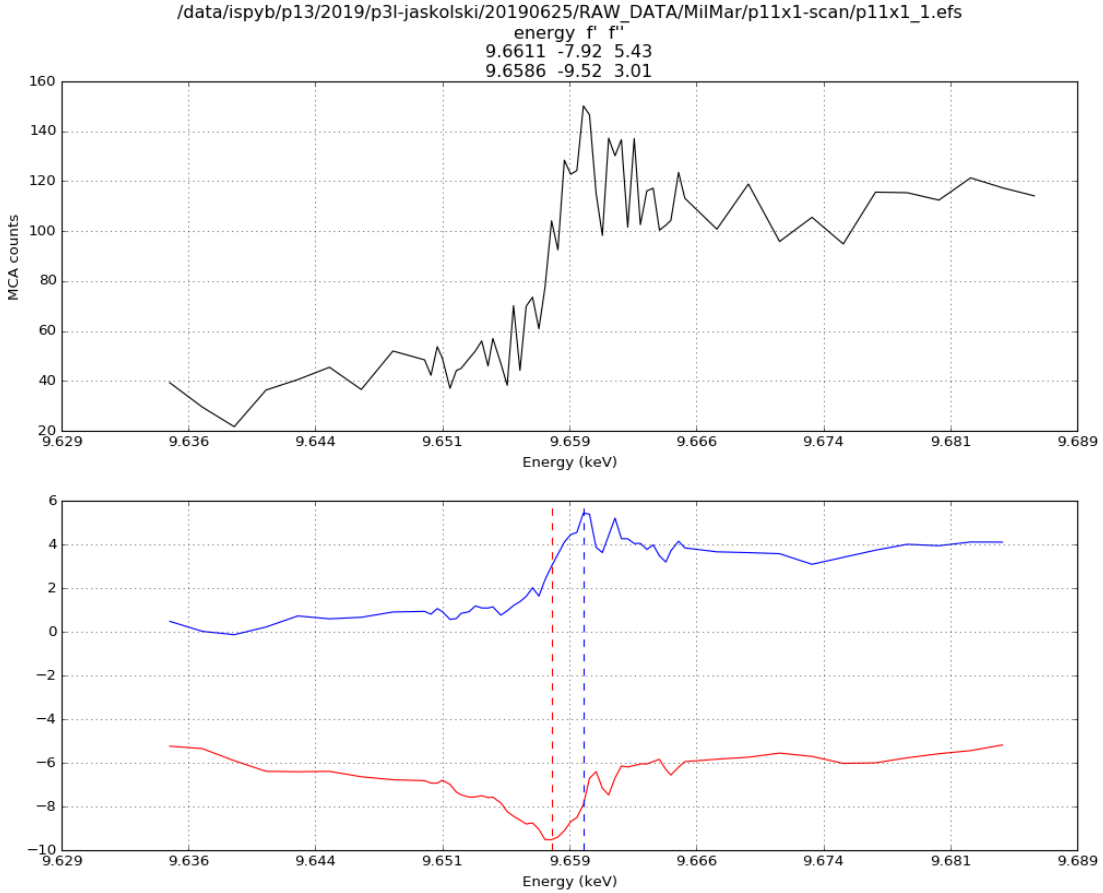

Supplementary Figure 9. X-Ray fluorescence spectrum recorded for ReAV crystal at Zn *K* absorption edge. The experimental spectrum is shown in the top panel (the theoretical value for Zn^0 is 9.6586 keV), and the corresponding f′ (red) and f″ (blue) curves are shown at the bottom panel. The vertical axis of the bottom panel is scaled in *e* units. Source data are provided as a Source Data file.

Supplementary References

Additional software used in this work:

- X-ray data collection: mxCUBE[1]
- Chromatography: Unicorn 5.31 (GE Healthcare)
- Circular dichroism: Spectra Manager (JASCO)
- NanoDSF: Prometheus software packages, PR.ThermControl v. 2.1.2, PR.ChemControl v. 1.4.1, PR.TimeControl v. 1.0.1. (NanoTemper)
- Isothermal titration calorimetry (ITC): MicroCal ITC 200 v. 1.26.0.1 (Microcal), MicroCal PEAQ-ITC Control Software v. 1.40.1319.0 (Panalytical)
- Differential scanning calorimetry (DSC): MicroCal PEAQ-DSC Software, v. 1.30 (MicroCal)
- Data analysis: Excel (MS Office 365) and Origin 7.0 (OriginLab).

References

1. Oscarsson, M. *et al.* MXCuBE2: the dawn of MXCuBE Collaboration. *J. Synchrotron Radiat.* **26**, 393–405 (2019).
