# Supplementary material for: Crystal structures of the elusive Rhizobium etlil-asparaginase reveal a peculiar active site
Source: Nat Commun. 2021 Nov 18;12:6717. doi: 10.1038/s41467-021-27105-x (PMC8602277; doi:10.1038/s41467-021-27105-x)
Supplement: Supplementary file 3 — Reporting summary [file 41467_2021_27105_MOESM3_ESM.pdf]

nature portfolio

Corresponding author(s): Mariusz Jaskolski

Last updated by author(s): Oct 23, 2021

Reporting Summary

Nature Portfolio wishes to improve the reproducibility of the work that we publish. This form provides structure for consistency and transparency in reporting. For further information on Nature Portfolio policies, see our Editorial Policies and the Editorial Policy Checklist.

Statistics

For all statistical analyses, confirm that the following items are present in the figure legend, table legend, main text, or Methods section.

n/a	Confirmed	
x		The exact sample size (*n*) for each experimental group/condition, given as a discrete number and unit of measurement
	x	A statement on whether measurements were taken from distinct samples or whether the same sample was measured repeatedly
x		The statistical test(s) used AND whether they are one- or two-sided<br>*Only common tests should be described solely by name; describe more complex techniques in the Methods section.*
x		A description of all covariates tested
	x	A description of any assumptions or corrections, such as tests of normality and adjustment for multiple comparisons
x		A full description of the statistical parameters including central tendency (e.g. means) or other basic estimates (e.g. regression coefficient) AND variation (e.g. standard deviation) or associated estimates of uncertainty (e.g. confidence intervals)
x		For null hypothesis testing, the test statistic (e.g. *F*, *t*, *r*) with confidence intervals, effect sizes, degrees of freedom and *P* value noted<br>*Give P values as exact values whenever suitable.*
x		For Bayesian analysis, information on the choice of priors and Markov chain Monte Carlo settings
x		For hierarchical and complex designs, identification of the appropriate level for tests and full reporting of outcomes
x		Estimates of effect sizes (e.g. Cohen's *d*, Pearson's *r*), indicating how they were calculated

Our web collection on statistics for biologists contains articles on many of the points above.

Software and code

Policy information about availability of computer code

Data collection	X-ray data collection: mxCuBE v2. Chromatography: Unicorn 5.31 (GE Healthcare). Circular dichroism (CD): Spectra Manager (JASCO). NanoDSF: Prometheus software packages, PR.ThermControl v. 2.1.2, PR.ChemControl v. 1.4.1, PR.TimeControl v. 1.0.1 (NanoTemper). Isothermal titration calorimetry (ITC): MicroCal ITC 200 v. 1.26.0.1 (Microcal), MicroCal PEAQ-ITC Control Software v. 1.40.1319.0 (Panalytical). Differential scanning calorimetry (DSC): MicroCal PEAQ-DSC Software, v. 1.30 (MicroCal).
Data analysis	Data processing and structure refinement: XDS (VERSION Jan 31, 2020 BUILT=20200417), Coot v.0.9.4.1 EL, CCP4 v.7.1.015, PyMOL v.1.7. Chromatography, CD, nanoDSF: Excel (MS Office 365), Origin 7.0 (OriginLab). DSC: MicroCal PEAQ-DSC Software, v. 1.30 (MicroCal).

For manuscripts utilizing custom algorithms or software that are central to the research but not yet described in published literature, software must be made available to editors and reviewers. We strongly encourage code deposition in a community repository (e.g. GitHub). See the Nature Portfolio guidelines for submitting code & software for further information.

Data

Policy information about availability of data

All manuscripts must include a data availability statement. This statement should provide the following information, where applicable:

- Accession codes, unique identifiers, or web links for publicly available datasets
- A description of any restrictions on data availability
- For clinical datasets or third party data, please ensure that the statement adheres to our policy

Atomic coordinates and structure factors corresponding to the final crystallographic models of ReAV generated in this study have been deposited in the Protein Data Bank (PDB) under accession codes 7os3 (START), 7os5 (OP), 7os6 (MP1), 7ou1 (MP2), and 7oz6 (MC). The corresponding raw diffraction images have been deposited in the Macromolecular Xtallography Raw Data Repository (https://mxrdr.icm.edu.pl) under DOI numbers: 10.18150/74YTYQ (START), 10.18150/MUKYJI

(OP), 10.18150/XBX6JE (MP1), 10.18150/VQQIHQ (MP2), and 10.18150/6W9HGI (MC). Source data are provided with this paper.

Field-specific reporting

Please select the one below that is the best fit for your research. If you are not sure, read the appropriate sections before making your selection.

- [x] Life sciences
- [ ] Behavioural & social sciences
- [ ] Ecological, evolutionary & environmental sciences

For a reference copy of the document with all sections, see nature.com/documents/nr-reporting-summary-flat.pdf

Life sciences study design

All studies must disclose on these points even when the disclosure is negative.

Sample size	No statistical method was used to determine sample size as it is not necessary for studies presented in the manuscript. Used samples were sufficient for all parameters determined in this study, e.g. biophysical measurements were repeated at least twice, while crystallographic data were collected for 2-3 crystals diffracting X-rays to the highest resolution and were analyzed to ensure the highest quality of the structural data.
Data exclusions	No data were excluded from the analysis.
Replication	Experiments were repeated at least 2-4 times as described in the manuscript. Biophysical measurements and crystallization experiments were performed for protein originating from different purification batches. All attempts at replication were successful.
Randomization	For structure refinement a subset of test reflections was selected at random for cross validation. No other randomization is needed for the studies described in the manuscript.
Blinding	All spectroscopic measurements (enzymatic activity, pH-dependent activity) were performed using control samples without addition of the enzyme. All samples, including controls, were analyzed in the same manner. No other blinding of samples is necessary for studies presented in the manuscript as data were derived form experiments with minimal risk of bias.

Reporting for specific materials, systems and methods

We require information from authors about some types of materials, experimental systems and methods used in many studies. Here, indicate whether each material, system or method listed is relevant to your study. If you are not sure if a list item applies to your research, read the appropriate section before selecting a response.

Materials & experimental systems

n/a	Involved in the study	
x		Antibodies
x		Eukaryotic cell lines
x		Palaeontology and archaeology
x		Animals and other organisms
x		Human research participants
x		Clinical data
x		Dual use research of concern

Methods

n/a	Involved in the study	
x		ChIP-seq
x		Flow cytometry
x		MRI-based neuroimaging
